# Editorial: Updates and Challenges in Maxillofacial Surgery

**DOI:** 10.3390/jcm14124345

**Published:** 2025-06-18

**Authors:** Andreas M. Neff

**Affiliations:** Department of OMFS, University Hospital Marburg and Philipps-University Marburg, 35043 Marburg, Germany; neffa@med.uni-marburg.de

## 1. Introduction

Oral and maxillofacial surgery (OMFS) is a rapidly evolving specialty that includes a wide variety of subspecialties, all of which are equally innovative in advancing OMFS. This Special Issue of JCM aimed to collate research discussing conceptual changes and improvements in the vast technical and surgical innovations introduced into the field of OMFS over recent years, such as virtual surgical planning, patient-specific implants, and regenerative techniques, just to name some of the most striking advances.

This S.O.R.G. Special Issue on “Updates and Challenges in Maxillofacial Surgery” has been made possible by a special educational research project funded by the Strasbourg Osteosynthesis Research Group (S.O.R.G.) between 2022 and 2024 and—to a major extent—consists of contributions from members of this international organization, in fact actively involving all sections of its European branch. As S.O.R.G., a non-profit organization, aims to promote scientific research and development, publishing findings in a recognized international journal such as *JCM* allows for some major goals of S.O.R.G. to be achieved, such as ongoing knowledge transfer through education at a global level and enhancing public awareness of medical advancements. The scientific independence of the contributing authors is strictly guaranteed by the principles which form the basis of the S.O.R.G.’s constitution. Being a part of S.O.R.G. involves engaging in scientific and technical activities aimed at developing and clinically assessing new techniques, devices, and therapies for craniomaxillofacial diseases, injuries, and disorders. S.O.R.G. members work to advance and complete specific research projects while also developing and enhancing quality control measures for treatment standards. Additionally, S.O.R.G. organizes continuous education and training events; evaluates and publishes scientific studies and research findings; and informs both the public and specialized groups through evidence-based, neutral public relation campaigns about the pros and cons of treatment options for craniomaxillofacial conditions. The organization also emphasizes the overall social significance of addressing these diseases at both the national and international levels and supports collaboration between local and international institutions involved in research and treatment evaluation. S.O.R.G. is organized into various scientific sections, which cover the whole field of Oral and Craniomaxillofacial Surgery, i.e., including the sections of oncology, reconstructive surgery, orthognathic surgery, facial plastic surgery, craniofacial surgery, TMJ, preprosthetic surgery, cleft palate, and trauma.

This editorial gives an overview of the contributions to these different sections, and some free papers related to the topic which have been successfully integrated into this S.O.R.G. Special Issue of *JCM* due to their high scientific quality.

## 2. Trauma

The S.O.R.G. trauma section members contributed three papers to this Special Issue. In their systematic review and meta-analysis “The Effectiveness of Three-Dimensional Osteosynthesis Plates versus Conventional Plates for the Treatment of Skeletal Fractures: A Systematic Review and Meta-Analysis” (2023), Raghoebar and coworkers from the S.O.R.G. trauma group point out that an adequate treatment of skeletal fractures is essential in trauma care [1], highlighting the potential benefits of using preformed anatomically shaped osteosynthesis plates and patient-specific implants (PSIs). These tools can enhance the accuracy of fracture reduction; offer greater stability; and—such as in oncology [2]—reduce the need for extensive surgical procedures and complications, as improper alignment of fractures can result in serious complications that negatively affect health and quality of life. Consequently, the systematic review aims to assess whether preformed anatomically shaped osteosynthesis plates and PSIs are more effective than traditional flat plates for treating skeletal fractures, particularly regarding anatomical reduction. The studies included were randomized clinical trials, prospective controlled trials, and both prospective and retrospective cohort studies with at least ten participants. Out of 5181 initially selected articles, 21 met the inclusion criteria. Notable differences in operation time were observed in the orbital (95% CI: −50.70–7.49, *p* = 0.008), upper limp (95% CI: −17.91–6.13, *p* < 0.0001), and lower limb groups (95% CI: −20.40–15.11, *p* < 0,00001). Additionally, the mean difference in the rate of anatomical reduction for the lower limp group favored the use of preformed anatomical plates and patient-specific implants over conventional plates (95% CI: 1.04–7.62, *p* = 0.04). In conclusion, the systematic review found a significant reduction in surgery time when using preformed anatomical plates and patient-specific implants for fractures in the orbital, upper, and lower limb extremities. Furthermore, these implants led to a higher rate of anatomical reduction in the lower limb compared to conventional flat plates [1].

The literature review and algorithm proposal by [3] and members of the S.O.R.G. trauma group analyzes airway management in cases of panfacial fractures evaluating various proposed methods for managing the airways, with an emphasis on controversies regarding these methods [4]. Additionally, it aims to provide a possible algorithm for effective airway management [3]. The proposed methods include, for example, orotracheal intubation (OTI), “front of neck access” (FONA), oral/nasal endotracheal intubation, submental intubation (SMI), or nasotracheal intubation (NTI). Obtaining and maintaining a secure airway in patients with panfacial fractures can be difficult. When assessing occlusion or when intermaxillary fixation is necessary, traditional airway devices cannot be inserted transorally unless placed in the retromolar region, which is not commonly practiced due to the challenges it poses for surgical access and fracture reduction. Submetal intubation (SMI), i.e., using the floor of the mouth for the passage of airway devices, offers a quite frequently practiced alternative. However, to minimize interference with occlusion, the use of nasotrachael intubation (NTI), by now, can be considered standard in managing facial fractures. Debates regarding NTI in cases of skull base fractures remain ongoing, but it has been proven safe when performed with fiberoptic guidance. Switching from NTI to an orotracheal tube (OTT) during surgery can help address, e.g., nasoorbitoethmoid fractures, although switching to OTT can be problematic in cases with significant soft tissue or mandibular/dentoalveolar injuries. Surgical tracheostomy is the preferred method for ensuring a clear airway in patients who require long-term intubation or multiple anesthetic procedures in a short time. Percutaneous tracheostomy can be an alternative to the traditional open tracheostomy and tends to have a slightly lower rate of complications, although it comes with some additional contraindications. Cricothyroidotomy is another option known for its low complication rate but is generally not used for long-term airway management in trauma cases. Staged surgery can help address some of these issues, focusing more on the careful planning and timing of various surgical interventions [3].

In their original paper, Pienkohs et al. (2023) from the Marburg working group analyze factors affecting the duration of surgery in the management of condylar head fractures. Although still controversially discussed, open reduction and internal fixation of condylar head fractures is becoming increasingly popular, particularly when instability is found in the vertical dimension, and even patients with multifragmented condylar head fractures have been reported to gain significant functional benefits from surgical treatment [5,6]. ORIF is particularly efficient in preventing post-traumatic occlusal disorders, which have been reported quite frequently for non-surgical treatment in condylar fractures [7]. Pienkohs et al. analyzed 168 cases of mandibular condylar head fractures treated surgically between 2007 and 2022, looking at surgery duration and various influencing factors. A bivariate analysis and linear regression revealed that the efficiency of muscle relaxation (*p* ≤ 0.001), type of fracture fragmentation (*p* = 0.031), and the age of the fracture (*p* = 0.003) are key factors affecting surgery duration, with the first surgeon’s experience being a constant factor. In conclusion, the authors, therefore, recommended that surgical intervention should occur as soon as reasonably possible after a traumatic event. Future studies should also establish a dosage regimen to enhance relaxation efficiency and take into account fragmentation type and associated fractures for a more precise estimation of operation time [8].

## 3. Orthognathic Surgery

The Heidelberg working group contributed to the orthognathic section, focusing on new techniques such as patient-specific plates for maxillary surgeries. By now, the approach of addressing significant skeletal deformities and malocclusion through a combination of orthodontic treatment and orthognathic surgery is well recognized and has been refined over the years [9]. Among the various surgical techniques developed, Le Fort osteotomies, in their different variations, are among the most commonly performed procedures in the field of oral and maxillofacial surgery. Two technological advancements have significantly impacted orthognathic surgery in recent years: 3D virtual planning and additive manufacturing. In their retrospective and monocentric cohort study “Accuracy of Patient-Specific Implants in Virtually Planned Segmental Le Fort I Osteotomies”, Kuehle et al. (2023) included patients who had received a virtually planned segmental maxillary osteotomy as part of their treatment. The positioning and fixation were accomplished using either a 3D-printed splint along with standard miniplates or patient-specific implants (PSIs). The study involved segmenting preoperative CT data, virtual planning data, and postoperative CBCT data. The analysis function of IPS CaseDesigner was used to measure how much each segment deviated from the intended position established during virtual planning. A total of 28 patients were in the patient-specific implant group, while 22 were in the conventional group. The PSI group demonstrated significantly less deviation from the planned position both anteroposteriorly (−0.63 ± 1.62 mm vs. −1.3 ± 2.54 mm) and craniocaudally (−1.39 ± 1.59 mm vs. −2.7 ± 3.1 mm). Additionally, the PSI group showed improved rotational deviations, with a better pitch (0.64 ± 2.59° vs. 2.91 ± 4.08°) and less inward rotation of the lateral segments. In conclusion, the findings indicate that patient-specific osteosynthesis was able to significantly minimize deviations from the preoperative plan in virtually planned surgeries, allowing for better management of transversal expansions and vertical positioning [9].

In their free-paper contribution, Hsiao and Fang from the National Cheng Kung University, Taiwan, presented an automatic voxel-based method for optimal symmetry plane generation for the maxillofacial region in severe asymmetry cases [10]. The symmetry of a person’s face is associated with their attractiveness and overall appearance. Generally, facial structures are symmetrical along the sagittal plane, which is essential for evaluating facial symmetry during aesthetic and reconstructive procedures. If the symmetry plane is not accurately determined, it can result in unsatisfactory surgical outcomes and may impact the mental health of patients [10]. The aim of their study was to create an automated and dependable approach for identifying the symmetry plane in the maxillofacial area. The authors evaluated a new method for determining the symmetry plane by comparing landmark- and surface-based approaches through quantitative assessments. A statistical analysis was used to check for significant differences among three types of symmetry planes. Twenty patients with severe facial asymmetry were reviewed. The findings indicated that the voxel-based method, termed the optimal symmetry plane (OSP), provides the most accurate representation of symmetry, with the OSP found to be significantly more symmetrical than those from the other two methods. The paired-voxel computation method introduced in this study can, therefore, be considered a strong and reliable way to identify the unique symmetry plane for patients with severe facial symmetry, which is important for surgical planning in orthognathic surgery [10].

## 4. Preprosthetic Surgery

The contribution of the preprosthetic section is a true state-of-the-art review by members of the S.O.R.G. preprosthetic section, involving a group of leading researchers in this field, focusing on the importance of modern preprosthetic surgery. Contemporary preprosthetic surgery aims to restore oral function and facial aesthetics in patients with tooth loss by using techniques such as bone grafting and dental implants, addressing issues such as alveolar bone loss, and improving denture stability, ultimately enhancing quality of life for affected individuals [11]. Patients with atrophic edentulous jaws often experience significant functional difficulties, leading to poor denture retention, a lower quality of life, and various health issues. The goal of preprosthetic surgery is to restore both function and appearance following tooth loss due to congenital issues, trauma, or surgical removal. Alveolar bone loss occurs because of disuse after teeth are lost. The introduction of dental implants, which help preserve bone, has transformed preprosthetic surgery. These implants can replicate the function of natural teeth, addressing the longstanding challenges of edentulism and jaw atrophy. The review also discusses various controversies in preprosthetic surgery, such as the debate between soft and hard tissue augmentation in aesthetic areas, bone regeneration versus prosthetic tissue replacement in the front of the maxilla, and the choice between sinus floor augmentation and short implants in the back of the maxilla, as well as the use of interpositional bone grafting versus only grafts for vertical bone augmentation [11].

## 5. Craniofacial Surgery

Once more, the Heidelberg working group contributed to the craniofacial section. Craniofacial surgery in infants can frequently lead to considerable blood loss during the procedure, necessitating significant intravenous fluid replacement, affecting homeostasis [12,13,14]. Due to their relatively small body volumes, infants are particularly vulnerable to fluctuations in their electrolyte balance. As a result, the type of fluid therapy used can influence their electrolyte levels and acid–base balance. In 2017, updated German national guidelines on perioperative infusion therapy were released, recommending the use of balanced, isotonic, and isoionic solutions. One group of infants received Sterofundin ISO^®^ before 2017 (based on the database of University Hospital Heidelberg), while another group received Deltajonin^®^ after the new guidelines were implemented. The main hypothesis of the study was that fluid resuscitation with a balanced electrolyte solution containing more than 120 mmol/L of chloride would result in higher rates of hyperchloremia and hyperchlomeric metabolic acidosis compared to a solution with less than 120 mmol/L of chloride. The study involved 100 infants who had craniectomy for isolated non-syndromic sagittal craniosynostosis. The first 50 infants received Sterofundin ISO^®^, while the next 50 received Deltajonin^®^ following updates to national guidelines in 2017. Both groups of infants were similar in terms of age, sex, underlying conditions, and preoperative electrolyte levels, with the exception of potassium (3.9 ± 0.3 mmol/L for Sterofundin ISO^®^ vs. 4.1 ± 0.3 mmol/L for Deltajonin^®^) and lactate (8.7 ± 2.1 mmol/L for Sterofundin ISO^®^ vs. 9.6 ± 2.6 mmol/L for Deltajonin^®^). In the Sterofundin ISO^®^ group, 19 patients experienced hyperchloremic metabolic acidosis, while only 2 infants in the Deltajonin^®^ group had this condition. The postoperative chloride levels were 111 ± 2.7 mmol/L for Sterofundin ISO^®^ compared to 108 ± 2.4 mmol/L for Deltajonin^®^. The anion gap was 12.5 ± 3.0 mmol/L for Sterofundin ISO^®^ and 14.6 ± 2.8 mmol/L for Deltajonin^®^, while the apparent strong-ion difference (SIDa) was 30.9 mmol/L for Sterofundin ISO^®^ and 33.8 mmol/L for Deltajonin^®^. Hyperchloremic acidosis can occur as a result of volume replacement using crystalloids that have a high chloride concentration, such as Sterofundin ISO^®^ [13].

Concerning craniofacial surgery, Kuehle et al. (2023) published the study “The Use of Artificial Intelligence for the Classification of Craniofacial Deformities” aiming to create a technological basis for a diagnostic tool that utilizes 2D images taken from personalized photogrammetric surface scans of patients [15,16]. The goal of this tool is to improve diagnostics in primary care and to reduce dependence on CT scans for young patients. Skull deformities occur in about 16% of newborns, while craniosynostosis is diagnosed much less frequently, at a rate of 0.05%. This condition involves the early fusion of one of the five skull sutures, which restricts normal growth of the neurocranium along the affected suture. In their retrospective cohort study, a total of 487 patients with photogrammetry scans were analyzed. The participants included 227 children with craniosynostosis, 206 with positional deformities and 54 healthy children. The study utilized fine-tuned ResNet-152 models during the training phase and performance was assessed using tenfold cross-validation. For detecting craionsynostosis, the model achieved a sensitivity of 0.94 and a specificity of 0.85. When differentiating among five specific classes (trigonocephaly, scaphocephaly, left positional plagiocephaly, right positional plagiocephaly, and healthy), sensitivity varied from 0.45 for left positional plagiocephaly to 0.95 for scaphocephaly, while specificity ranged from 0.87 for right positional plagiocephaly to 0.97 to scaphocephaly. The authors explain that a larger dataset would be necessary to identify rarer types of craniosynostosis. The 2D approach also allows for future applications using digital cameras or smartphones [16].

## 6. Temporomandibular Joint Surgery

The S.O.R.G. TMJ group contributed two papers, alongside two free papers. In their study titled “Controversial Aspects of Diagnostics and Therapy of Idiopathic Condylar Resorption: An Analysis of Evidence- and Consensus-Based Recommendations Based on an Interdisciplinary Guideline Project”, the Marburg working group provides a comprehensive analysis of the complexities involved in diagnosing and treating idiopathic condylar resorption (ICR), a rare condition characterized by osteolysis of the mandibular condylar process [17]. ICR is a condition primarily seen in women aged 15 to 35, with incidence related to maxillofacial surgeries varying widely, from 1% to 31%. The initial literature search yielded 97 records, and after a detailed selection process, 56 relevant studies were included in the updated guidelines. The international literature, particularly from Anglo-American sources, often cites orthognathic surgeries as significant contributing factors. Based on an interdisciplinary evidence-based guideline project utilizing the Delphi method, the authors identified key areas of controversy, such as initial imaging techniques, pharmacotherapy, and the timing of surgical interventions. The guideline emphasizes that CT/CBCT is the current standard for initial imaging, while MRI may offer additional insights, particularly regarding soft tissue assessment. Despite these advancements, certain recommendations remain open due to insufficient evidence, and several key aspects of idiopathic condylar resorption (ICR) remain to be discussed, highlighting the complexity of its diagnosis and treatment. The study also emphasizes the unclear etiology of ICR and suggests that further research is needed to clarify potential risk factors, particularly related to orthognathic surgery [17].

The Amsterdam group contributed a CAE report and review of the literature on hemifacial hyperplasia (HFH), a rare congenital condition that involves significant overgrowth of hard and soft tissues on one side of the face, leading to noticeable facial asymmetry [18,19]. It is typically present at birth and continues to grow proportionally until the individual reaches adulthood, after which the growth ceases. A condition that falls under the spectrum of hemifacial hyperplasia is congenital infiltrating lipomatosis of the face (CIL-F). The authors explain that only a few reports have been published on CIL-F affecting the face, particularly cases where the condition is linked to bony ankylosis of the TMJ. The purpose of the study was to present a case of CIL of the face in which the patient also exhibited signs of unilateral condylar hyperplasia and associated ankylosis of the TMJ. This occurred years after the patient had undergone a condylectomy, at the age of seventeen. Five years later, the patient experienced ankylosis of the TMJ associated with congenital CIL, which resulted in progressively limited mouth opening and facial pain in the temporal region. In this patient with CIL, successful TMJ reconstruction was carried out using an alloplastic total joint prosthesis [20,21,22]. One year after the surgery, the patient achieved optimal mouth opening, complete relief from temporal facial pain, and stable dental occlusion. The TMJ prosthesis successfully restored joint function and significantly enhanced the patient’s quality of life. Future studies will be necessary to explore the long-term outcomes of TMJ reconstruction in patients with congenital infiltrating lipomatosis of the face (CIL-F) [18].

In a free-paper contribution, Kazuya Yoshida (2023) investigated superior dislocations of the condyle into the middle cranial fossa, which require invasive procedures for repair in the absence of a timely diagnosis [23,24,25]. The study analyzed 104 articles involving 116 patients, with a mean age of 25.7 years (±15.9 years), ranging from 5 to 81 years, and a median age of 22 years. Among the patients, 78 were females (67.7%) and 38 were males (33.3%), and 60% of the affected females and 87.5% of the affected males required open reduction. The ratio of closed to open procedures within the first 7 days post-injury remained consistent; however, the use of closed reduction procedures declined over time, with all cases necessitating open reduction after 22 days. In total, 80% of patients with complete intrusion of the condyle needed open reduction, while the rates for both procedures were similar in other cases. Open reduction was performed significantly more often in males (*p* = 0.026, odds ratio: 4.959, 95% confidence interval: 1.208–20.365) and less frequently in cases of partial intrusion (*p* = 0.011, odds ratio: 0.186, 95% confidence interval: 0.051–0.684). The frequency of procedures also varied based on the time until treatment (*p* = 0.027, odds ratio: 1.124, 95% confidence interval: 1.013–1.246). Based on these results, Yoshida (2023) emphasizes the importance of an effective diagnostic imaging and a collaborative approach involving oral surgeons, neurosurgeons, and otolaryngologists. These subjects are essential for the timely diagnosis and minimally invasive treatment of superior condylar dislocation, thus avoiding long-term complications [25].

In another free-paper contribution, [26] present a systematic review on the effectiveness of administering I-PRF in TMJ cavities for the treatment of TMDs, with eight studies included overall in the review [26]. Five of the included studies are randomized controlled trials (RCTs) involving 213 patients. In each of the RCT groups, arthrocentesis was performed, and 1–2 mL of I-PRF (centrifuged at 700 rpm for 3 min a 60× *g*) was administered per joint. After three months, articular pain decreased to 0–25% of the initial pre-intervention levels in the I-PRF groups, compared to 38–50% in the control groups. Mandibular mobility improved to 121–153% in the I-PRF groups and 115–120% in the control groups. The main limitations of the evidence included the small number of RCTs and the absence of any RCT groups that received I-PRF without prior arthrocentesis. In conclusion, according to the systematic review, administering I-PRF when rinsing the temporomandibular joint further alleviates pain and enhances mandibular mobility. The findings of this meta-analysis, therefore, indicate that administering I-PRF alone may have a positive impact on TMJ functioning, leading to a reduction in joint pain, limitations in mandibular movement, and the severity of acoustic symptoms. Additionally, studies that compare the outcomes of I-PRF treatment with and without arthrocentesis, particularly regarding the regenerative effects of this preparation on TMJ structures, are considered beneficial [26].

## 7. Oncology and Reconstructive Surgery

The S.O.R.G. reconstructive group was highly active, contributing four publications. Zittel et al. presented a prospective multi-center study with the title “Impact of Salvage Surgery on Health-Related Quality of Life in Oral Squamous Cell Carcinoma” [27]. Recurrence of oral squamous cell carcinoma (OSCC) occurs in up to 50% of patients with advanced primary tumors, regardless of the initial treatment, especially when the surgical resection is challenging due to the anatomy [28]. Health-related quality of life is closely linked to tumor progression. The standard treatment for advanced OSCC is primary surgery followed by chemoradiotherapy. However, if the cancer recurs, treatment options are limited. Re-radiation has dose restrictions due to tissue toxicity, especially in early recurrences, and the radiation dose is often already maximized from the initial treatment. Therefore, salvage surgery is the preferred option if a cure is intended. The requirement for significant ablative surgery along with microvascular reconstruction suggests that the treatment is both invasive and painful, and the functional results may be uncertain. To evaluate how salvage surgery affects the health-related quality of life (HRQoL) of patients with recurrent OSCC, a multi-center prospective study was launched. A total of 55 patients participated in the study, with an average follow-up period of 26.7 months. After 12 months, patients with stage 1 and 2 recurrent tumors reported better global health status scores (60.83) compared to their baseline scores (53.33). In contrast, patients with advanced recurrent tumors showed only slight improvements in their global health scores after 12 months (55.12) compared to a baseline (53.2). Regarding orofacial pain, patients with small recurrent tumors experienced a significant decrease in pain levels after salvage surgery, with scores dropping from 41.67 at baseline to 20.37 after 12 months. For advanced recurrent tumors, a notable reduction in pain was observed three months post-surgery, with scores decreasing from 47.76 at baseline to 29.7. However, swallowing function was assessed as worse at the 12-month mark compared to a baseline for all tumor stages, with mean scores indicating a decline in function. Based on these results, [27] conclude that the HRQoL can remain stable even after significant surgical procedures. Specifically, salvage surgery appears to positively influence pain management. Since swallowing function may be affected post-surgery, reconstructive methods should prioritize the anatomy related to swallowing, and functional therapies should be actively included in early rehabilitation efforts [27].

Reconstructing complex midface deformities using vascularized free-tissue transfers after cancer surgeries is another significant challenge addressed by [29]. Traditionally, microvascular connections for free flaps have relied on cervical vessels, which require access to the neck and can lead to external scars and longer pedicle lengths [30,31]. To address these issues, this study explores the use of intraoral anastomoses through facial vessels as a promising alternative. A total of 117 patients underwent procedures involving 132 flaps, which included 91 osseous and 41 cutaneous flaps. The preparation of facial vessels inside the oral cavity was completed successfully in under one hour, with no complications from the dissection or connections. In two instances, the vessel diameter was too small for connections, requiring an external approach. Over a follow-up period of 48 months, two osseous flaps were lost, leading to a 1.5% loss rate among the 132 flaps. Additionally, three flaps had partial losses, including a skin island from a scapula, a femur border zone, and a rectus muscle flap, resulting in a 2.3% partial loss rate from the 130 flaps used. This case series highlights the practicality of using intraoral anastomoses for immediate complex reconstruction of the midface after cancer surgery [29].The animal study by [32] investigates the impact of transfer-related ischemia on free flap metabolism and electrolyte homeostasis [32]. The study aims to explore whether transfer-related ischemia affects the metabolism and electrolyte levels within a flap, in comparison to central venous blood, following the transfer of free flaps in pigs. The results of this study show that there is considerable tissue damage caused by interruptions in blood flow related to transfer, which occurs after a brief period of ischemia. This leads to the activation of anaerobic glycolysis, an increase in lactate levels, and acidosis, which ultimately results in ion imbalances and cell death. To prevent permanent tissue damage, it is crucial to minimize the duration of ischemia. Additionally, the effectiveness of the newly developed animal model has been confirmed, allowing it to be used in future experiments that aim to explore the molecular mechanisms involved in thrombus formation in free flaps [23,33].

Finally, the prospective monocentric trial by [34] focuses on the cancer-related outcomes and the quality of life for patients with early-stage oral squamous cell carcinoma after they underwent surgical removal of the tumor and reconstruction using free-tissue flaps [34]. The main goals of the study were to assess overall survival, progression-free survival and quality of life for up to 24 months post-surgery. A total of 26 patients participated in the study. The results showed a 100% overall survival rate and a 92.3% progression-free survival rate, with a maximum follow-up of 21 months. There were no significant changes in overall quality of life after the surgery. However, patients did report a notable decrease in pain (*p* = 0.048) and less difficulty with speech one year after the procedure (*p* = 0.021). The free-flap reconstruction method proved to be safe and led to excellent oncological outcomes and quality of life. The study effectively shows that free-flap reconstruction for early-stage oral squamous cell carcinoma is a safe and effective approach that yields strong oncological outcomes while maintaining a good quality of life for patients [34].

As the section editor of this Special Issue focusing on updates and challenges in OMFS, I do sincerely hope that by integrating research from the different S.O.R.G. scientific sections, this collection of papers has provided a holistic overview, not only with regard to innovations such as patient-specific hardware and up-to-date planning but also to address problems that must be resolved to further advance this specialty and improve the outcomes and benefits for our patients.
